# Epidemiology of Malaria in Northern Madagascar as Exemplified by the Mampikony District

**DOI:** 10.3390/pathogens14090848

**Published:** 2025-08-26

**Authors:** Daria Kołodziej, Wanesa Wilczyńska, Daniel Kasprowicz, Małgorzata Marchelek-Myśliwiec, Krzysztof Korzeniewski

**Affiliations:** 1 Department of Epidemiology and Tropical Medicine, Military Institute of Medicine–National Research Institute, 128 Szaserów St., 04-141 Warsaw, Poland; dkolodziej@wim.mil.pl (D.K.); wanesa.wilczynska@gmail.com (W.W.); kkorzeniewski@wim.mil.pl (K.K.); 2 Clinique Medicale Beyzym, Ambatoboeny District, Manerinerina 403, Madagascar; daniel.kasprowicz@icloud.com; 3 Clinic of Nephrology, Transplantology and Internal Medicine, Pomeranian Medical University, 72 Powstańców Wielkopolskich Av., 70-111 Szczecin, Poland

**Keywords:** malaria, *Plasmodium*, mRDT, RT-PCR, Madagascar

## Abstract

Malaria is a parasitic disease caused by *Plasmodium* protozoa, which is a serious public health issue in Madagascar, an island country located off the coast of Africa in the Indian Ocean. Despite significant efforts to prevent the spread of communicable diseases, the country’s epidemiological situation is worrying and has been deteriorating in recent years, mostly due to poverty and limited access to healthcare. The aim of the present study was to assess the prevalence rates of malaria in northern Madagascar, as exemplified by the Mampikony District, between 2023 and 2024, as well as to assess the effectiveness of the methods used for malaria diagnosis. Material and methods. The study was conducted on a sample of 782 local residents who reported to healthcare centres in Mampikony between 2023 and 2024. The methods used to establish the diagnosis of malaria included rapid diagnostic tests (mRDTs) and molecular assays (RT-PCRs). Results. The 2023 study conducted on a sample of 484 patients demonstrated malaria prevalence of 4.5% (by mRDTs) and 8.2% (by RT-PCR), while the 2024 study carried out on a sample of 298 patients demonstrated malaria prevalence of 8.4% (by mRDTs) and 12.4% (by RT-PCR). The analysis of demographic variables showed that malaria was more prevalent in women and in adults; however, the differences between individual study groups were not statistically significant. In this study, positive malaria cases were predominantly caused by *P. falciparum*, but we also found cases caused by *P. vivax* as well as mixed infections. Conclusions. The study results support the need to apply more sensitive diagnostic tools for malaria diagnosis, e.g., RT-PCR. Also, our findings indicate the necessity to reassess and update the strategies for the treatment of malaria in the region due to a growing asymptomatic malaria carriage. To control the spread of malaria in Madagascar, it is essential to apply a wide range of interventions.

## 1. Introduction

Malaria is a parasitic tropical disease caused by protozoa of the *Plasmodium* genus. The disease remains a serious public health issue in many tropical and sub-tropical countries, especially in the countries lying in Sub-Saharan Africa, where *Anopheles mosquitoes*, i.e., infection vectors, breed best. *P. falciparum* is the predominant *Plasmodium* species in Africa, and it is responsible for most malaria cases on this continent. Other *Plasmodium* species, such as *P. vivax*, *P. ovale*, and *P. malariae*, have also been documented to cause disease in humans, yet they are much less common than *P. falciparum* [1,2].

Madagascar is one of the lowest-income countries in the world. Its residents face numerous limitations in access to diagnostic and medical services [3,4], which is one of the reasons for exceptionally high rates of infectious and parasitic diseases in the country. Lack of political stability, weaknesses in the functioning of public institutions, and poor governance create significant obstacles for economic growth, while the tropical climate, which is characterized by high temperatures and significant rainfall (during the wet season lasting from November until April), creates ideal breeding conditions for numerous vectors which can transmit infectious diseases. The regions with the worst epidemiological situation include rural and forested areas, as well as lowlands. According to the World Bank, 81% of people in Madagascar live below the poverty level, i.e., on less than $1.90 per day [5]. The country’s poor economic situation, and especially a lack of resources at a local level, is a major obstacle to combating many diseases, including malaria. The National Strategic Plan 2018–2022 called for progressive malaria elimination beginning in low-incidence districts (<1 case/1000 population). The goal of the plan was to reduce malaria incidence by at least 30%, and malaria-related mortality by 50% [6]. However, the outbreak of the COVID-19 pandemic, which intensified the economic problems of the country and changed the national healthcare priorities, disrupted malaria control interventions. As a result, the intended goal has not been achieved [7]. In fact, quite the opposite has been observed: the incidence of malaria in Madagascar has increased from 1 to 100 cases per 1000 population since 2021 [8]. In 2023, the overall number of malaria cases in Madagascar reached 2.8 million, with an estimated 400 malaria-related deaths, which was a significant increase from 1.7 million cases reported in 2022 [9]. To effectively combat malaria, efforts must consider the epidemiological situation in the region, drug resistance mechanisms, as well as the effectiveness of locally adopted preventive measures. It is equally important to take into account the diversity of *Plasmodium* species responsible for malaria in humans. Currently, most malaria control strategies focus on the elimination of *P. falciparum*, which is responsible for most malaria cases. However, it must be kept in mind that other *Plasmodium* species capable of causing malaria in humans (including mixed infections) are also prevalent in East Africa. Thus, interventions which focus solely on the identification and elimination of *P. falciparum* only are ineffective and result in an underestimation of malaria incidence in the region. In Madagascar, malaria incidence peaks during the rainy season; however, transmission also occurs during the dry season, but at a lower rate. The fact that asymptomatic malaria cases are detected throughout the year suggests that local people serve as a reservoir for *Plasmodium* species; this contributes to the continued presence of the disease and seasonal outbreaks. Understanding malaria prevalence is essential for adapting malaria control strategies to local transmission dynamics [10]. The aim of the present study was to investigate the prevalence rates of malaria during the low transmission season, with a particular focus on asymptomatic infections; the study was conducted in the Mampikony region, northern Madagascar, over two consecutive years.

## 2. Materials and Methods

### 2.1. Study Group

**Figure 1:**
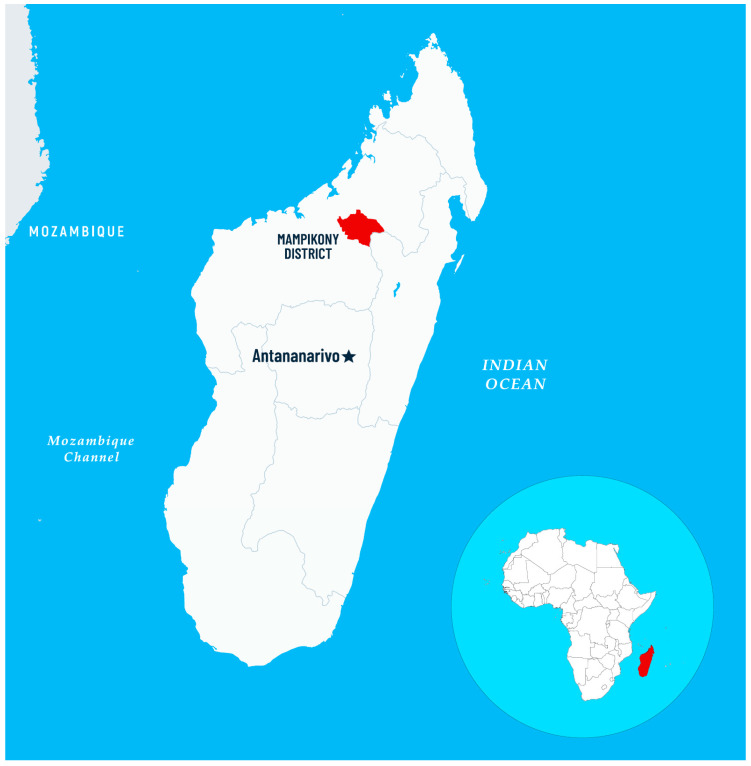
Map of Madagascar with the location of Mampikony District.

The study was conducted on a sample of patients recruited from among the residents of the Mampikony District, Northern Madagascar (a commune inhabited by 28.5 thousand people, which lies at 55 m above sea level, at geographic coordinates approximately 16°06′ S 47°38′ E). The region is characterized by a varied topography from lowlands to wetlands, with most local people living in rural areas (Figure 1). Tropical climate with a pronounced rainy season, which lasts from November until April, high temperatures, and heavy rainfall provide favorable conditions for the reproduction and spread of *Plasmodium* parasites. The country has a high proportion of young population, especially children and adolescents. The local economy is predominantly agrarian, with most inhabitants engaged in farming, livestock raising, or seasonal work. Many local people have limited access to education and struggle with the inability to afford healthcare services. It is worth emphasizing that a substantial proportion of the population lives below the poverty line, which also hinders access to health care.

Each person who volunteered to take part in the study was informed of the study purpose and was requested to sign an informed consent form to participate in the study. In case of paediatric patients, informed consent was provided by a child’s parent or their legal guardian. Patient information sheets had been translated into Malagasy language, and all procedures were explained to the participants by qualified personnel. In 2023, biological samples were collected from randomly selected students attending local schools (a state-owned school and a private school) as well as from randomly selected people living in villages across the Mampikony District (*n* = 484). The study involved both students from local schools as well as residents of local villages, thus making the sample more representative in terms of age and background of the participants. In 2024, biological samples were collected from randomly selected residents of the Mampikony District (*n* = 298) who reported to Clinique Beyzym—a healthcare centre located in the Manerinerina commune and volunteered to participate in the study. In 2024, out of 298 study participants, 254 were children under the age of 15 (85.2%), while in 2023, 418 out of 484 participants (86.4%) were in this age group. The study consisted of 2 stages. The first stage of the study was conducted in Madagascar. During this stage, study participants were screened for malaria using mRDTs; next all mRDT-positive results were confirmed by light microscopy to exclude potential false positives. This is consistent with the diagnostic practices commonly used in Madagascar, where access to other methods, such as molecular diagnostics, is limited. Following this, a sample of venous blood was collected from each of the study participants and applied onto Whatman FTA cards for further diagnostics. The second stage of the study was carried out in Poland, where molecular tests were performed. This allowed for a more precise analysis of malaria prevalence and the identification of *Plasmodium* species. All tests (both in 2023 and 2024) were performed between June and July, i.e., during the dry season when malaria incidence is reportedly low. According to the UNICEF report, in 2023, malaria peak occurred in December, i.e., during the rainy season [11].

### 2.2. Malaria Screening

#### 2.2.1. Malaria Rapid Diagnostic Tests (mRDT)

The research study consisted of two stages. During the first stage, malaria rapid diagnostic tests (mRDTs) were performed on venous blood samples, which had been collected by qualified medical staff. Next, the medical staff recorded selected demographic variables of the study participants (age and sex), conducted patient interviews and recorded any symptoms reported by the patients (fever, headaches, chills, sweats, muscle pain, nausea) as well as any information regarding the use of antimalarial medication within 6 months prior to the commencement of the study. Following this, a sample of 200–300 µL venous blood was collected from each participant, and the samples were applied onto Whatman FTA cards (Whatman, Maidstone, UK). The cards were left to dry (to obtain dried blood spots), and next, they were transported to Poland for further testing. Quick tests for malaria were performed using a lateral flow chromatographic immunoassay NADAL^®^ Malaria Pf/Pan Ag 4 species (nal von minden GmbH, Regensburg, Germany). The test can be used for a simultaneous, qualitative detection and differentiation of two markers: HRP II (histidine-rich protein II) specific to P. falciparum and pLDH (lactate dehydrogenase) specific to all Plasmodium species. According to the information provided by the manufacturer, the test’s sensitivity and specificity are both 99.1%.

#### 2.2.2. Molecular Diagnostics RT-PCR

Molecular diagnostics was performed at the Department of Epidemiology and Tropical Medicine of the Military Institute of Medicine–National Research Institute in Poland (DETM MIM-NRI) on dried blood spot samples (DBS). Real-time polymerase chain reaction (RT-PCR) was used to identify and differentiate between the four most prevalent Plasmodium species (i.e., *P. falciparum*, *P. vivax*, *P. malariae*, *P. ovale*). First, four discs 2 mm in size were punched out from the Whatman FTA cards with a Harris Uni-Core punch (Qiagen, Hilden, Germany), and the discs were placed in 1.5 mL Eppendorf tubes. Next, genetic material was extracted from the samples using the Sherlock AX Kit (A&A Biotechnology, Gdańsk, Poland) in accordance with the manufacturer’s instructions. The procedure for the isolation of genomic DNA works on the principle of nucleic acid absorption on ion-exchange membranes, combined with DNA precipitation with isopropanol. The extracted genetic material was suspended in 100 µL TE elution buffer and stored at the temperature of −20 °C until further tests. A commercial Bosphore Malaria Genotyping Kit v1 (Anatolia Geneworks, Istanbul, Turkey) set, which is used for qualitative detection of *P. falciparum*, *P. malariae*, *P. ovale*, and *P. vivax* DNA, was used to perform RT-PCR. This kit uses the gene encoding 18S ribosomal RNA (rRNA) as a target for detecting Plasmodium parasites. RT-PCRs were run on AriaMx RT-PCR system (Agilent Technologies, Santa Clara, CA, USA) following the thermal protocol recommended by the manufacturer (Table 1).

### 2.3. Statistical Analysis

The data analyses were performed using an Excel spreadsheet (Microsoft Corporation, Redmond, WA, USA). Mean, median, minimum, and maximum values were calculated. Categorical variables included sex (male/female), age group (<5, 5–14, >15 years), year of data collection (2023/2024), and malaria test result (positive/negative). To assess associations between categorical variables and malaria infection status, the chi-square test of independence was used. Specifically, we tested for statistical associations between infection status and age group, sex, and year of study. The threshold for statistical significance was set at *p* = 0.05. Statistically non-significant findings, such as the lack of association between sex and infection status, were not discussed further in the main text.

### 2.4. Ethical Approval

Our research project was granted approval by the Ministry of Public Health in Antananarivo, Madagascar (Ref. No. 256–23/MSANP of 3 July 2023) and (no. 108-24/MSANP/SPC of 5 April 2024).

## 3. Results

A total of 782 residents of the Mampikony District in Northern Madagascar were tested (with 484 people tested in 2023 and another 298 in 2024). Children under the age of 14 years old (mean age 10.8, median: 9) accounted for a majority of the study participants—in 2023 they accounted for 82% of all study subjects (mean age: 11.4, median: 10), and in 2024 for 89% of the participants (mean age: 8.4, median: 5). In 2023, participant ages ranged from 1 to 60 years, while in 2024, the age range was from 1 to 81 years. Overall, there were more female than male participants [427 women (54.6%) vs. 355 men (45,4%)]. On the day of the test, 54/782 (6.9%) participants reported fever [19 people (3.9%) in 2023 and 35 people (11.7%) in 2024], and 6/782 (0.8%) reported the use of antimalarial medication in the last 6 months before the study (3 people in 2023 and 3 people in 2024). The demographic variables of the study participants and their medical data are presented in Table 2.

The tests performed in 2023 showed that 5.6% of all study subjects were infected with *Plasmodium falciparum* (no other *Plasmodium* infections were confirmed) (Table 3). In 2024, we observed a more than double increase in the number of *Plasmodium* positive cases (12.4%) (Table 4). In addition, apart from *P. falciparum* infections, we also found two cases of *P. vivax* infection and one case of a mixed infection with *P. falciparum + P. vivax*. In 2023, no statistically significant difference was found between malaria prevalence and sex (*p* = 0.326); in 2024, we observed that the number of positive malaria cases was nearly twice as high in women as in men (*p* = 0.039). It was also observed that the number of positive cases was higher in adults and adolescents over 15 years old than in smaller children; however, this difference was not statistically significant (*p* = 0.085). In 2023, none of the 19 positive malaria cases reported fever. In contrast, in 2024, 11 out of 35 (31.4%) individuals who had reported fever were found to be malaria positive by RT-PCR. Also, out of six people reporting the use of antimalarials within the past 6 months, four people were found to have *Plasmodium* in their blood.

The study demonstrated that, in 2024, malaria prevalence was more than 2-fold higher compared to 2023 (12.4% vs. 5.6%), and the difference in malaria prevalence between these 2 years was found to be statistically significant (*p* = 0.001). While this finding represents a key observation and may suggest a potential deterioration in the epidemiological situation, it should be interpreted with caution, taking into account the study’s limitations—including its observational nature, the seasonality of transmission, and potential confounding factors.

## 4. Discussion

The 2023 World Malaria Report released by the World Health Organization (WHO) said that of an estimated 263 million malaria cases globally, a rise of 11 million compared to the previous year. In Madagascar, the reported number of malaria cases in 2023 exceeded the national epidemic threshold: 2.8 million cases [9]. This upward trend continued in 2024. In fact, in the first 19 weeks of 2024, a total of 1,531,902 malaria cases and 212 malaria-related deaths were reported from Madagascar. This clearly shows that there is an urgent need to prioritize malaria control and prevention efforts in the region [12].

Poor reporting and ineffective diagnostic systems are significant obstacles to effective malaria prevention in Madagascar. Malaria reports prepared by local healthcare providers are not reliable as they often contain inaccurate information, are often incomplete, and show a low uptake of malaria treatment [13]. In Madagascar, rapid diagnostic tests (mRDTs) are the primary tool for diagnosing malaria. Although these tests are affordable and readily available, they have some serious limitations, including a high rate of false negative results. In this study, a double band (Pf/Pan bands) was present on 17 positive mRDTs; the presence of a double band may be associated with a higher parasite density (parasitaemia) or an infection by a *Plasmodium* species other than *P. falciparum*. However, malaria rapid diagnostic tests (RDTs) are not capable of distinguishing between different *Plasmodium* species. Simultaneous detection of both HRP2 and pLDH antigens in a malaria rapid diagnostic test (mRDT) can result in the appearance of a double band. HRP2 protein is specific to *P. falciparum*, while pLDH is present in all human-infecting *Plasmodium* species. The presence of a double band on a malaria test may also be indicative of a mixed *P. falciparum* and *P. vivax* infection, or it may be a result of a nonspecific test reaction—occasionally observed with elevated antigen levels—which can activate both test lines. This observation has been reported in various settings and does not necessarily reflect high parasite density. In our 2023 study, of the 27 samples which tested positive on RT-PCR, only 10 had tested positive on mRDTs. In 2024, rapid tests confirmed 25 malaria cases, whereas the molecular methods showed 37 positive cases.

An increasing prevalence of submicroscopic malaria infections is yet another obstacle to effectively controlling malaria in Madagascar. Although this type of malaria infection has not been well documented, there are some studies that suggest that it may be a serious health problem in some African countries. The results of the 2014 study by Howes et al. [14], which was conducted in southern Madagascar, showed that most of the identified malaria cases were asymptomatic or submicroscopic. There have been many reports [15] which suggest that molecular diagnostic techniques are much more accurate and reliable than rapid diagnostic tests, especially in settings with high rates of asymptomatic or submicroscopic malaria carriage. Expanding the application of RT-PCR testing in tropical and subtropical countries can help generate reliable epidemiological data, assess the infection dynamics, and implement more effective interventions for malaria control. Unfortunately, widening access to molecular testing in resource-limited settings is prevented by its high cost. WHO recommended an ambitious plan for malaria control and prevention, “Global Technical Strategy for Malaria 2016–2030”. The first pillar of this plan is to ensure universal access to malaria prevention, diagnosis, and treatment; this, however, may be hard to achieve, especially in Africa, where access to diagnostic and treatment services is limited [16]. Our findings are in line with the results of studies by other authors, which reported that the number of malaria cases in Africa, including in Madagascar, has been on the rise since 2023. To effectively control and eliminate malaria, a comprehensive approach is necessary, encompassing effective control of a variety of vectors and *Plasmodium* species, strengthening of the healthcare system infrastructure, ensuring universal access to malaria diagnosis and treatment, as well as improving diagnostic and treatment capabilities [17].

Limited access to healthcare facilities, especially in rural areas of Madagascar, is a significant problem, particularly during the rainy season (which is the peak malaria transmission period) when transportation is severely impacted. Natural disasters can also disrupt access to essential medical services by causing significant damage to the healthcare sector infrastructure. In 2008, for example, 167 outpatient clinics and 6 hospitals were damaged by extreme weather events [18]. Apart from the above, healthcare providers in Madagascar are also struggling with a shortage of skilled professionals, a lack of supplies, and financial difficulties. Economic problems mean that public hospitals are often incapable of providing recommended treatment, with patients often receiving symptomatic treatment only, which can manage the symptoms but is insufficient to address the underlying infection [9].

Fever is the main symptom of malaria, but it is also a common sign of many other tropical diseases, making it difficult to differentiate from other conditions and often resulting in incorrect diagnosis. For this reason, it is essential that all febrile cases suspected of being malaria should be confirmed by laboratory testing before malaria treatment is given [19]. Asymptomatic malaria infections are highly prevalent in Madagascar, and this fact has been confirmed by the results of our 2023 and 2024 studies, which demonstrated that only some of the patients who tested positive for malaria exhibited the classic malaria signs and symptoms. In 2023, a total of 27 people tested positive for malaria, but only 19 of those people (3.9% of the whole study group) had symptoms suggestive of malaria; in 2024, there were 37 confirmed malaria cases, with 35 of those (11.7%) reporting malaria symptoms.

The diversity of *Plasmodium* species, which can cause malaria in humans, is yet another challenge in the fight against the disease. While *P. falciparum* is the most common cause of severe malaria and is responsible for most deaths, other species of *Plasmodium*, including *P. vivax*, *P. malariae,* and *P. ovale*, are also found in the African population and can cause disease. These species have unique life cycles and therefore require specific treatments. More importantly, *P. vivax* and *P. ovale* have the ability to form hypnozoites (a dormant stage of the parasite) in the liver cells, which can cause a relapse after months or years of the initial infection. Furthermore, the increasing incidence of *P. vivax* malaria in Duffy-negative individuals (i.e., individuals who lack the Duffy antigen, i.e., a receptor used by *P.vivax* on their red blood cells) who were previously considered resistant to malaria, suggests a need for improved diagnostics for this species [20]. In 2024, two cases of malaria caused by *Plasmodium vivax* and one case of a mixed malaria infection (*P. falciparum* + *P. vivax*) were reported from the Mampikony District, Madagascar. This finding supports the need for more accurate diagnostic methods to be used in the region, especially in view of a growing asymptomatic malaria carriage in Madagascar [21].

Another worrying issue and a challenge in the fight against malaria is the high incidence of malaria in the paediatric population. Children are disproportionately affected by severe malaria, including its cerebral form, and are therefore at a higher risk of complications or death. Malnutrition, which is widespread in the Mampikony District, additionally weakens children’s immune system, making them more susceptible to various infections [17]. While prevention is crucial for malaria control, its implementation is often hampered by economic factors [22]. Despite the implementation of the National Malaria Control Program (in cooperation with the CDC), which consists of the distribution of insecticide-treated nets (ITNs), the distribution of mRDTs, indoor residual spraying (IRS0), and prevention of malaria in pregnancy, the situation of malaria has deteriorated in recent years [23]. Due to economic conditions and lifestyle in many regions of Madagascar, women and men spend a comparable amount of time outdoors, both at work and in daily activities. Local communities largely rely on agriculture and small-scale outdoor tasks, regardless of gender. The literature has not identified clear and systematic differences in malaria infection rates between women and men, which is consistent with the results of this study. Infections occur at similar levels among both sexes, suggesting that under comparable environmental conditions and exposure, the risk of malaria transmission is similar. Furthermore, there are studies indicating that most infections occur outside the home, which further confirms that lifestyle and place of activity have greater significance than gender [24,25]. The example of Madagascar shows how difficult it is to effectively control malaria in endemic regions, despite significant prevention efforts and the implementation of the recommended malaria control strategies. Studies have shown that not all prevention methods are equally effective. For example, indoor residual spraying (IRS) using non-pyrethroid-like insecticides has been shown to be more effective in reducing malaria incidence in Madagascar than insecticide-treated bed nets (ITNs) [26]. In addition, a growing insecticide resistance in vector species and the emergence of new mosquito species, which contribute to the spread of malaria, compromise the effectiveness of the interventions that are commonly used for malaria prevention and control. *Anopheles gambiae*, which is the main malaria vector on the eastern and western coast of Madagascar, contributes to high transmission rates of the disease year-round [27,28]. Also, researchers have recently observed the emergence of new vectors on the island, such as *Anopheles coustani*, which also plays a role in malaria transmission [29]. Notably, the expansion of new mosquito species has been observed in other parts of Africa as well, e.g., the expansion of *Anopheles stephensi* in Ethiopia, Sudan, Nigeria, Ghana, and Kenya. This particular species, which is native to South Asia and the Arabian Peninsula, has been seen to thrive in urban environments. The expansion of the newly emerging species in cities and their migration to non-transmission areas poses a significant threat to populations lacking natural immunity to malaria.

Madagascar is becoming an increasingly popular tourist destination, mostly because of its unique national parks. For this reason, although it may seem isolated, the island is not immune to colonization by new mosquito species, including those species that are vectors of newly emerging malarial species. The introduction of new malaria vectors, such as *Anopheles stephensi*, can significantly disrupt existing malaria epidemiology, posing substantial challenges to control efforts [30]. To reduce malaria transmission into new areas, a multi-pronged approach is needed, including intensifying preventative measures, adopting new diagnostic and treatment methods, and adapting malaria control strategies to the changing epidemiological situation.

## 5. Limitations of the Study

There were several limitations to this study. These included limited access to comprehensive demographic and health-related data, which would allow us to evaluate the nutritional status of the participants and to determine the prevalence rate of malnutrition in the study sample (malnutrition is a significant risk factor and a predictor for malaria, especially in children). Further, we lacked information on patients’ comorbidities, which could have affected their overall health status and the severity of signs and symptoms. There were several limitations to this study. These included limited access to comprehensive demographic and health-related data, which would allow us to evaluate the nutritional status of the participants and to determine the prevalence rate of malnutrition in the study sample (malnutrition is a significant risk factor and a predictor for malaria, especially in children). Further, we lacked information on patients’ comorbidities, which could have affected their overall health status and the severity of signs and symptoms. Another important limitation was the failure to perform microscopic examination (using the thick blood smear) of all the samples to validate the mRDT results. In addition, the study timing must be considered when interpreting the findings–the study was conducted during the dry season, which is outside the peak malaria transmission season. For this reason, the results may not fully represent the epidemiological situation in the region. Future studies should be conducted using more advanced and accurate diagnostic tools, and they should be carried out during the rainy season when malaria prevalence is typically the highest. A significant limitation of the study is the marked predominance of children among participants, which prevents a reliable analysis of the relationship between Plasmodium infections and demographic variables such as age and sex. Due to the lack of significant differences between sexes and the small number of adults in the sample, it was not possible to draw meaningful conclusions in this regard.

## 6. Conclusions

The study findings show an increase in the number of malaria cases in the Mampikony region between 2023 and 2024, this difference is likely due to the different settings in which data were collected—hospital-based recruitment in 2024 and community-level screenings in schools and villages in 2023—which may suggest a higher malaria prevalence among hospitalized individuals than in the general population. Climatic factors and the quality of healthcare services may also influence the malaria situation in the region. Diagnostic challenges, particularly the use of rapid diagnostic tests (mRDTs), and a rise in the number of asymptomatic infections are hindering accurate disease identification. The use of molecular assays, such as RT-PCR, can improve malaria detection rates; however, it may affect the feasibility of large-scale implementation. Further, the presence of *Plasmodium vivax* infections alongside *P. falciparum* malaria in the study participants necessitates adapting diagnostic and therapeutic strategies to include all malarial species. To address the emerging threats associated with the geographical expansion of new mosquito species, including *Anopheles stephensi*, and to maintain effective malaria control in the region, malaria surveillance and control programs must be enhanced.

## Figures and Tables

**Table 1:** Thermal protocol for RT-PCR.

Cycles	Phase	Temperature [°C]	Time [min]
1	Polymerase activation	95	14:30
50	Denaturation	97	00:30
	Annealing (data collection)	60	01:00
1	Hold	32	05:00

**Table 2:** Demographic variables and medical data of the study participants.

	2023–2024		2023		2024	
Total	782		484		298	
	N	%	N	%	N	%
Gender						
Female	431	55.1	277	57.2	154	51.7
Male	351	44.9	207	42.8	144	48.3
Age						
<5	182	23.3	76	15.7	106	35.6
5–14	481	61.5	323	66.7	158	53.0
>15	119	15.2	85	17.6	34	11.4
Medical Data						
Symptoms	54	6.9	19	3.9	35	11.7
Antimalarial drugs	6	0.8	3	0.6	3	1.0

**Table 3:** The number of *Plasmodium* infections detected by mRDT vs. RT-PCR in the sample of Malagasy people involved in the 2023 study by age and gender (Mampikony District, *n* = 484).

Variables		mRDT			RT-PCR	*p*-Value
		Pf	Pf + Pan	Total	*P. falciparum*	
		N (%) [95% Cl]	N (%) [95% Cl]	N (%) [95% Cl]	N (%) [95% Cl]	
Total	484 (100%)	8 (1.7) [0.5; 2.8]	2 (0.4) [0.0; 1.0]	10 (2.1) [0.8; 3.3]	27 (5.6) [3.5; 7.6]	
Gender						0.326 ^1^
Female	277(57.2%)	4 (1.4) [0.0; 2.8]	1 (0.4) [0.0; 1.1]	5 (1.8) [0.2; 3.4]	13 (14.3) [2.2; 7.2]	
Male	207 (42.8%)	4 (1.9) [0.1; 3.8]	1 (0.5) [0.0; 1.4]	5 (2.4) [0.3; 4.5]	14 (6.8) [3.3; 10.2]	
Age						0.022 ^1^
<5	76 (15.7%)	0 (0.0) [0.0; 0.0]	0 (0.0) [0.0; 0.0]	0 (0.0) [0.0; 0.0]	4 (5.3) [0.1; 10.3]	
6–14	323 (66.7%)	6 (1.9) [0.4; 3.3]	0 (0.0) [0.0; 0.0]	6 (1.9) [0.4; 3.3]	13 (4.0) [1.9; 6.2]	
15–60	85 (17.6%)	2 (2.4) [0.0; 5.6]	2 (2.4) [0.0; 5.6]	4 (4.8) [0.2; 9.2]	10 (11.8) [4.9; 18.6]	

Pf—*Plasmodium falciparum*; Pan—*Plasmodium* antigen, 95%CI—95% confidence interval; ^1^—chi-squared test.

**Table 4:** The number of *Plasmodium* infections detected by mRDT vs. RT-PCR in the sample of Malagasy people involved in the 2024 study by age and gender (Mampikony District, *n* = 298).

Variables		mRDT			RT-PCR				*p*-Value
		Pf	Pf + Pan	Total	*P. falciparum*	*P. vivax*	*P. falciparum* *+ P. vivax*	Total	
		N (%) [95% Cl]	N (%) [95% Cl]	N (%) [95% Cl]	N (%) [95% Cl]	N (%) [95% Cl]	N (%) [95% Cl]	N (%) [95% Cl]	
Total	298 (100%)	10 (3.4) [1.3; 5.4]	15 (5.0) [2.6; 7.5]	25 (8.4) [5.2; 11.5]	34 (11.4) [7.8; 15.1]	2 (0.7) [0.0; 1.6]	1 (0.3) [0.0; 1.0]	37 (12.4) [8.7; 16.2]	
Gender									0.039 ^1^
Female	154 (51.7%)	8 (5.2) [1.7; 8.7]	8 (5.2) [1.7; 8.7]	16 (10.4) [5.6; 15.2]	22 (14.3) [8.8; 19.8]	2 (1.3) [0.0; 3.1]	1 (0.7) [0.0; 1.9]	25 (16.3) [10.4; 22.1]	
Male	144 (48.3%)	2 (1.4) [0.0; 3.3]	7 (4.9) [1.3; 8.4]	9 (6.3) [2.3; 10.2]	12 (8.3) [3.8; 12.8]	0 (0.0) [0.0; 0.0]	0 (0.0) [0.0; 0.0]	12 (8.3) [3.8; 12.8]	
Age									0.910 ^1^
<5	106 (35.6%)	0 (0.0) [0.0; 0.0]	5 (4.7) [0.7; 8.8]	5 (4.7) [0.7; 8.8]	12 (11.3) [5.3; 17.4]	1 (0.9) [0.0; 2.8]	0 (0.0) [0.0; 0.0]	13 (12.4) [6.0; 18.5]	
6–14	158 (53.0%)	8 (5.1) [1.6; 8.5]	8 (5.1) [1.6; 8.5]	16 (10.2) [5.4; 14.8]	17 (10.8) [5.9; 15.6]	1 (0.6) [0.0; 1.9]	1 (0.6) [0.0; 1.9]	19 (12.0) [6.9; 17.1]	
15–81	34 (11.4%)	2 (5.9) [0.0; 13.8]	2 (5.9) [0.0; 13.8]	4 (11.8) [0.9; 22.6]	5 (14.7) [2.8; 22.6]	0 (0.0) [0.0; 0.0]	0 (0.0) [0.0; 0.0]	5 (14.7) [2.8; 22.6]	

Pf—*Plasmodium falciparum*; Pan—*Plasmodium* antigen; 95%CI—95% confidence interval; ^1^—chi-squared test.

## Data Availability

The data presented in this study are available on request from the corresponding author.
